# Human Norovirus Surrogate Is Highly Stable in Berry Smoothies and under In Vitro Simulated Digestion

**DOI:** 10.3390/foods13071066

**Published:** 2024-03-30

**Authors:** Riya Hooda, Malak A. Esseili

**Affiliations:** Center for Food Safety, Department of Food Science and Technology, University of Georgia, Griffin, GA 30223, USA

**Keywords:** human norovirus, Tulane virus, foodborne outbreaks, strawberry, blueberry, in vitro digestion, INFOGEST

## Abstract

Human noroviruses are major causes of foodborne outbreaks linked to berries. The overall goal of this study was to investigate the persistence of a human norovirus surrogate, Tulane virus (TV), in berry smoothies and under simulated digestion through the gastrointestinal track. Two types of smoothies were prepared from blueberries and strawberries. Tulane virus was spiked into each smoothie and incubated either at 37 or 4 °C for 2, 60, and 120 min. Furthermore, the virus-spiked smoothies were subjected to sequential oral (2 min), gastric (10 and 60 min), and intestinal (15 and 120 min) digestion according to the standardized INFOGEST model. Quantification of infectious TV was carried out using the TCID_50_ assay. At 4 °C, in both berry smoothies, TV infectivity did not show significant changes throughout the 120 min period. At 37 °C, TV infectivity showed significant reduction (~0.5 log TCID_50_/mL) only in blueberry smoothies starting at 60 min. During the oral, gastric, and intestinal digestion phases, the mean log reduction in TV infectivity in blueberry did not exceed ~0.5 log, while infectious TV in strawberry smoothies under all phases was stable. Given the notable stability of infectious viruses in berry smoothies and the gastrointestinal tract, prevention of norovirus contamination of berries is paramount to reduce virus outbreaks linked to berries.

## 1. Introduction

Human norovirus (HuNoV) is the most common cause of diarrheal disease in all age groups, resulting in about one-fifth of the cases and 200,000 deaths worldwide annually [1,2]. In the United States of America, HuNoV incidences are highest in children under the age of 5 and in adults above the age of 65 [3]. Infections with HuNoV cause gastrointestinal symptoms (diarrhea, vomiting, stomach pain, and nausea), which are usually self-limiting in healthy adults, but can be severe and prolonged in young children, the elderly, and immuno-compromised patients [4]. As a foodborne pathogen, HuNoV is the leading cause of foodborne illnesses in the USA, causing an estimated 58% of all the cases [5], and resulting in direct and indirect estimated costs of USD 2 billion [6]. It is mainly transmitted via the fecal–oral route and the contamination of food and water. Historically, the lack of efficient approaches to culturing HuNoV in cell lines limited the ability to devise control strategies that prevent norovirus foodborne outbreaks. Surrogate culturable viruses, such as murine norovirus (MNV) and Tulane virus (TV), are often used to gain insights into HuNoV inactivation under various interventions, with TV being preferred to study HuNoV persistence in food and in the environment, because it binds to the histo-blood group antigens similar to HuNoV [7,8,9].

Produce at risk of contamination with HuNoV includes salad crops such as lettuce and other leafy greens and soft fruits such as berries. The contamination of these commodities with HuNoV can occur at any stage along the farm-to-fork chain, via a number of sources, including contaminated water used for irrigation or processing and asymptomatically HuNoV-infected food harvesters or food handlers who do not follow proper hygiene practices [10,11]. Globally, over a period of 35 years (1983 to 2018), there were 94 berry-associated outbreaks that resulted in ~23,000 illnesses [12]. Examining these outbreaks showed that the majority (80%) were caused by foodborne viruses, with HuNoV being the most common causative agent associated with over 46 outbreaks [12]. Berries can be consumed fresh without cooking (such as in fruit smoothies), and, if they are contaminated, they may pose an elevated health risk to consumers.

Multiple studies reported the detection of foodborne viruses in berries sampled from different countries. For example, the prevalence of foodborne viruses ranged between no detection (Italy), 6.7% (France), 9–12% (China), to as high as 34% (Belgium) [13,14,15]. The detection of foodborne viruses on berries is not surprising, because previous studies have shown that HuNoV (or its surrogate MNV) persisted for days to weeks on the surface of berries, depending on the storage temperature and berry type [16,17,18,19]. For example, the infectivity titer of MNV on the surface of strawberries was reduced by 0.7 log after 7 days at 4 °C; however, a similar log reduction occurred after 2 days at 25 °C [17]. In contrast, on blueberries, MNV was much more stable, showing 0.66 after 21 days at 4 °C [16]. These two previous studies used different methods to recover the viruses from blueberries and strawberries, and their results cannot be directly compared. However, it seems that these viruses were more stable on blueberry surfaces in comparison to strawberries. In a direct comparison between viruses spiked on the surface of raspberries and those on strawberries, it was observed that a faster decay of MNV occurred on strawberries in comparison to raspberries (1 vs. 3 days to achieve 1 log reduction, respectively) [19]. On frozen berries, HuNoV was shown to remain infectious for at least 3 months [18]. These studies suggested that the detection of viruses on berries can lead to infections in consumers and foodborne outbreaks, partially due to the long persistence of foodborne viruses on berries, which often exceeded the shelf life of fresh berries. This assumption is likely complicated by other factors such the level of virus contamination and the type of berries. For example, some berries are reported to have antiviral properties (reviewed in [20]) that were shown to inactivate MNV [21]. Furthermore, heathy volunteers who consumed berries that were later found to be contaminated with relatively low levels of HuNoV (Ct values 32.9–39.7) did not report any clinical symptoms (vomiting or diarrhea) [22]. There is a knowledge gap concerning the persistence of HuNoV inside berry matrices and after contaminated berries are consumed and digested. Therefore, the overall objective of this study was to assess the persistence of a HuNoV surrogate, TV, in berry smoothies (homogenates made with water) and under simulated in vitro digestion. We used the standardized INFOGEST model of digestion in which the composition of the digestive fluids simulates the oral, gastric, and intestinal tract pH, chemical composition, and digestive enzymes [23].

## 2. Materials and Methods

### 2.1. Tulane Virus Stock Preparation and the TCID50 Infectivity Assay

**Figure 1:**
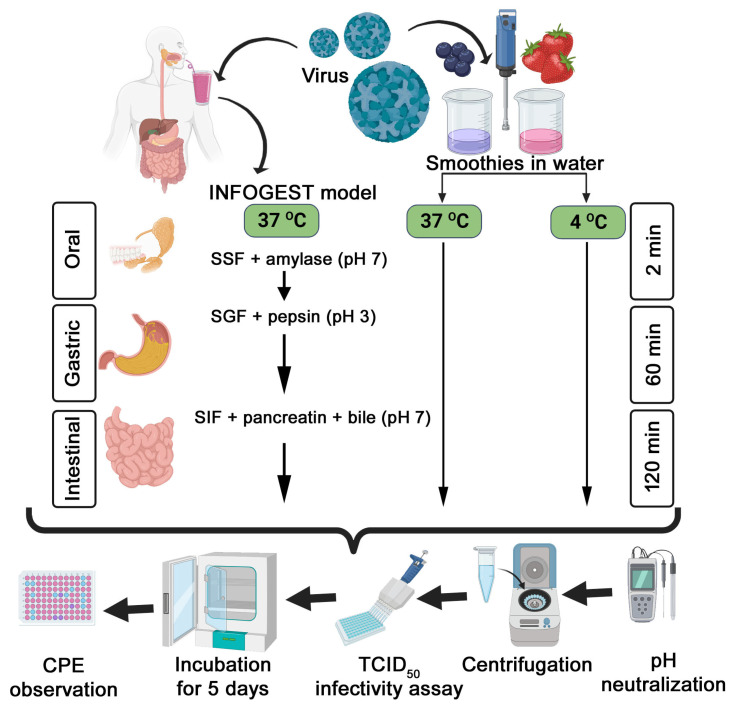
A flowchart depicting the general experimental design of this study. The flowchart was created using BioRender.com.

The TV stock was generated in the LLC-MK2 cell line (ATCC CCL-7, Gaithersburg, MD, USA) as described in our previous publications [8,9]. The stock virus was ultrafiltered using Amicon 100 KDa Ultra-15 centrifugal devices (Millipore, MA, USA) to semi-purify the virus from cell culture lysate and further concentrate the virus. The TCID_50_ assay was performed as described in our previous publications [8,9]. The stock TV titer was ~6.5 log TCID_50_/mL. The general experimental design of this study is presented in Figure 1.

### 2.2. TV Survival in Berry Smoothies

Fresh blueberries and strawberries were bought from local grocery stores. To prepare the smoothies, berries (50 g) were gently washed, cut into small pieces, and then blended in sterile water (at a ratio of 1:1 *w*/*v*) using the Hamilton Beach 2-speed Hand Blender (Walmart, GA, USA). The berry smoothies were then aliquoted into 1.5 mL Eppendorf microcentrifuge tubes to which TV was added at a ratio of 1:10 (*v*/*v*). The pH of the berry smoothies and the volume of 2.5 M NaOH required to neutralize the pH were determined for each experiment using berry smoothie samples containing no viruses. The average pH of blueberry and strawberry smoothies was 2.8 ± 0.2 and 3.3 ± 0.05, respectively. Samples were incubated at 4 °C or 37 °C for a period of 2, 60, 120, and 240 min. For positive controls, the virus was added to sterile water only, while negative controls were composed of berry smoothie samples with no added viruses. At the end of incubation periods, the berry smoothie samples were neutralized, and centrifuged at 12,000 rpm for 5 min at 4 °C, as described previously [24]. The supernatants were transferred to new tubes and 1% antibiotic–antimycotic cocktail (Fisher Scientific, Waltham, MA, USA) was added to inhibit bacterial or fungus growth. Samples were immediately quantified using the TCID_50_ assay on the LLC-MK2 seeded in 96-well plates.

### 2.3. Simulated In Vitro Digestion of TV Spiked into Berry Smoothies

The Simulated Salivary Fluids (SSFs), Simulated Gastric Fluids (SGFs), and Simulated Intestinal Fluids (SIFs) were prepared as described in the standardized INFOGEST protocol [23]. The pH of the SSF, SGF, and SIF was adjusted to 7, 3, and 7, respectively, as described previously [23]. The simulated fluids were supplemented with digestive enzymes as described in our previous publications [25,26]. Briefly, the human salivary α-amylase was added to SSF, porcine pepsin was added to SGF, and porcine pancreatin was added to SIF at a final concentration of 75 U/mL, 8 mg/mL, and 5 mg/mL, respectively. Also, SIF was supplemented with porcine bile at a final concentration of 2 mg/mL. The in vitro digestion fluids were supplemented with the enzymes, bile, and CaCl_2_ (H_2_O)_2_ just prior to their use in the experiments. All chemicals and enzymes were purchased from Sigma-Aldrich (Burlington, MA, USA).

Three consecutive phases of simulated digestion, oral alone (2 min), gastric (5 and 60 min), and intestinal (15 and 120 min), were performed on TV spiked into sterile water and berry smoothies, respectively. The berry smoothies were prepared by blending them as described above in SSF at a ratio of 1:1 (*w*/*v*). The samples were incubated at 37 °C. For samples in the gastric phase and proceeding to the intestinal phase, the pH of the samples was neutralized before the addition of intestinal fluids. Following the end of each digestion period, the pH of the berry samples was neutralized using 2.5 M NaOH, and the volume of the samples was adjusted by adding sterile water to achieve equal volumes for all the samples in each digestion phase. The samples were centrifuged at 12,000× *g* rpm for 5 min at 4 °C. The supernatants were transferred to Eppendorf microcentrifuge tubes supplemented with 1% antibiotic–antimycotic cocktail and tested using TCID_50_ assay to quantify the virus titers.

### 2.4. Statistics Analyses

GraphPad Prism version 5 (GraphPad Software, San Diego, CA, USA) was used for all statistical analyses. Experiments were independently repeated three times, and three technical replicates were tested for each treatment or time point. The entire dataset was transformed to log_10_. The log reductions in infectivity titers were calculated based on virus infectivity titers initially recovered from each matrix (water, blueberry, or strawberry smoothies). One-way or two-way analysis of variance (ANOVA) followed by Tukey or Bonferroni post-tests, respectively, were used to determine significant differences in mean infectivity titers. The factors analyzed included time, temperature, and matrix. Differences in means were considered significant when the *p*-value was less than 0.05 and were denoted in the figures by different letters. Data were expressed as the mean ± standard error (SE).

## 3. Results

### 3.1. Survival of Infectious TV Spiked into Berry Smoothies

**Figure 2:**
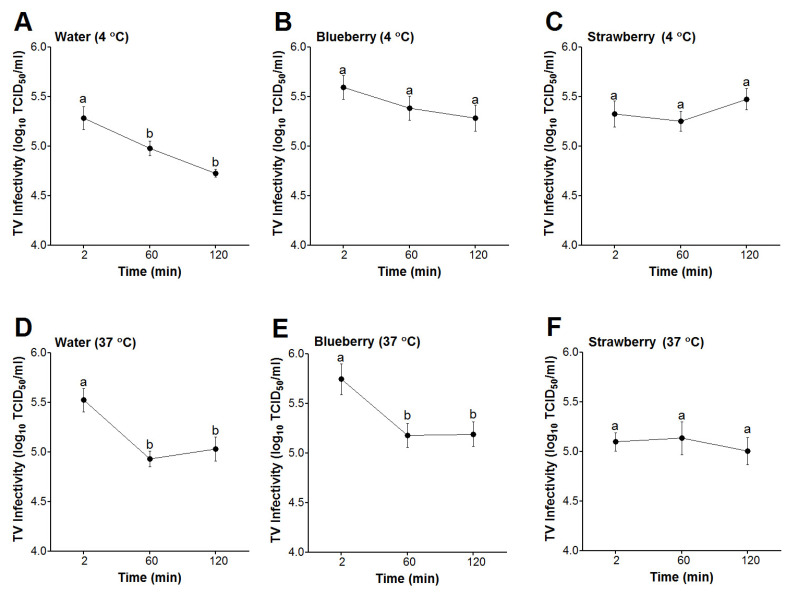
Survival of infectious TV in berry smoothies. The virus was spiked in water, blueberry, and strawberry smoothies at 1:10 (*v*/*v*) and then incubated for 2, 60, or 120 min at 4 °C (**A**–**C**) or 37 °C (**D**–**F**). Error bars indicate standard error. Means with different letters indicate significant differences (*p* < 0.05).

At 4 °C, the infectivity titers of TV in sterile water showed significant decreases at the 60- and 120-min incubation periods in comparison to TV titers at 2 min (Figure 2A). However, the infectivity titers of TV spiked into blueberry or strawberry smoothies showed non-significant changes over the same time points (Figure 2B,C). Similarly, at 37 °C, TV infectivity titers in water showed significant decreases at the 60- and 120-min time points in comparison to the virus titers at 2 min (Figure 2D). However, at this temperature, TV infectivity titers in blueberry smoothies showed significant decreases at the 60- and 120-min period in comparison to TV titers in blueberries at 2 min (Figure 2E). In contrast, TV spiked into strawberry smoothies and incubated at 37 °C showed no significant changes throughout the 120 min incubation period (Figure 2F). For all matrices and at both temperatures tested, there were no significant changes in TV infectivity titers between the 60- and 120-min incubation periods (Figure 2). Overall, our results suggested that infectious TV remained stable in strawberry smoothies at both 4 and 37 °C throughout the 120 min period.

### 3.2. Time and Temperature Effects on TV Infectivity Log Reductions in Berry Smoothies

**Figure 3:**
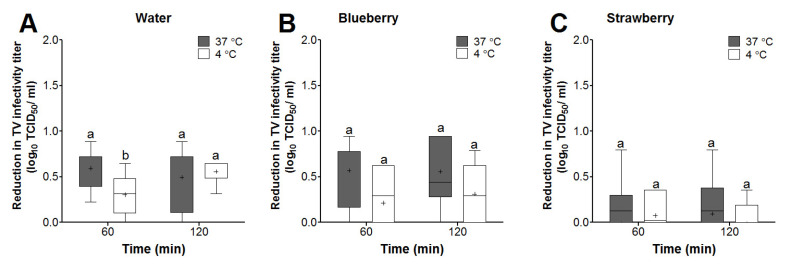
Box and whisker plots showing the effect of time and temperature on TV infectivity reductions (log TCD_50_/mL) in (**A**) water and (**B**) blueberry and (**C**) strawberry smoothies. The virus infectivity log reductions were determined with respect to recovered virus titers at the 2 min time point for each matrix. Boxes show the 25th percentile, median, 75th percentile, and mean (+), while whiskers show the maximum and minimum values. Means with different letters indicate significant differences (*p* < 0.05).

Reductions in TV infectivity titers at the 60- and 120-min time points were calculated with respect to the 2 min TV titers for each matrix at each temperature. In sterile water, only the mean log reduction in TV infectivity titers at 60 min was significantly different between the 37 and 4 °C incubation temperatures (0.59 vs. 0.30 log reduction, respectively) (Figure 3A). Two-way ANOVA analyzing time and temperature as factors revealed that time and temperature alone were not significant factors affecting TV log reductions in water. However, the interaction between time and temperature was a significant factor, explaining 11.3% of the variation in the TV log reduction data in water.

In blueberry smoothies, the mean TV infectivity log reductions were higher at 37 as compared to 4 °C for both the 60- and 120-min time points (0.56 and 0.55 vs. 0.21 and 0.31 log reductions, respectively); however, this change was not statistically significant (Figure 3B). Two-way ANOVA analyzing time and temperature as factors revealed that only temperature was a significant factor, explaining 15% of the variation in the TV log reduction data in blueberry homogenates.

In strawberry smoothies, the mean log reductions for TV (<0.1 log) were not significant for any time point under any temperature tested (Figure 3C). Two-way ANOVA revealed that time, temperature, or the interaction between these two factors had no significant effects on the changes in TV log infectivity titers in strawberry smoothies.

Comparing the various matrices, the mean log reductions for TV in blueberry smoothies were not significantly different from that in water at all temperatures and time points tested (Figure 3A,B). However, the mean log reductions for TV in blueberry smoothies were significantly higher than those observed in strawberry smoothies at all temperatures and time points tested (Figure 3B,C). Taken together, these results suggested that temperature alone is a significant factor affecting TV reduction in blueberry smoothies, while temperature or time had no significant effects on TV in strawberry smoothies.

### 3.3. Survival of Infectious TV Spiked in Berry Smoothies under Simulated In Vitro Digestion

**Figure 4:**
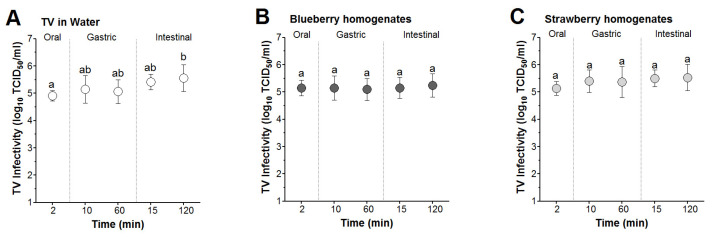
In vitro simulated digestion of TV spiked into berry smoothies. The virus was spiked in (**A**) water and (**B**) blueberry and (**C**) strawberry smoothies at 1:10 (*v*/*v*) and then incubated at 37 °C under the oral (2 min), gastric (10 and 60 min), and intestinal phases (15 and 120 min). Error bars indicate standard deviation. Means with different letters indicate significant differences (*p* < 0.05).

TV spiked into sterile water and blueberry and strawberry smoothies was subjected to in vitro simulated digestion consisting of consecutive exposures to oral (2 min), gastric (10 and 60 min), then intestinal (15 and 120 min) fluids. For TV spiked in sterile water, the mean infectivity titers at the end of the oral phase were not significantly different from TV titers at the end of the 10 and 60 min gastric or 15 min intestinal digestion phases (Figure 4A). However, the mean TV infectivity titer at the end of the 120 min intestinal phase was significantly different than that at the 2 min oral phase (5.5 vs. 4.9 log, respectively) (Figure 4A). For TV spiked in blueberry and strawberry smoothies, the mean infectivity titers for TV showed non-significant changes throughout the oral, gastric, and intestinal digestion phases (Figure 4A–C). Taken together, these results suggested that infectious TV was stable in blueberry and strawberry smoothies when subjected to oral, gastric, and intestinal fluids simulating digestion at 37 °C for 2, 60, and 120 min, respectively.

### 3.4. Time and Matrix Effects on TV Infectivity Log Reductions following In Vitro Digestion

**Figure 5:**
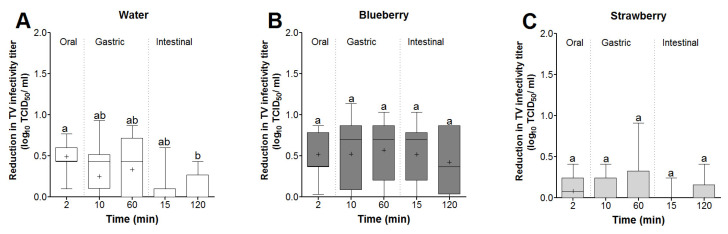
Box and whisker plot showing the effect of in vitro digestion on TV infectivity log reduction. Virus log reductions in (**A**) water and (**B**) blueberry and (**C**) strawberry smoothies were determined based on the 2 min virus titers for each matrix. Boxes show the 25th percentile, median, 75th percentile, and mean (+), while whiskers show the maximum and minimum values. Means with different letters indicate significant differences (*p* < 0.05).

The reductions in TV infectivity were calculated with respect to the mean virus titer recovered from water and blueberry and strawberry smoothies at the 2 min time points (5.4, 5.6, and 5.2 log TCID_50_/mL, respectively). In water, the highest TV mean log reduction occurred during the oral phase, which was not significantly different from the reduction occurring during the 60 min gastric phase (0.49 vs. 0.33 log reduction, respectively) (Figure 5A). However, the intestinal phase showed the lowest mean reduction in TV infectivity (<0.01 log), which was significantly lower than the oral phase (Figure 5A). In blueberry smoothies, the mean log reduction in TV infectivity at the oral phase was not significantly different from that at the 60 min gastric or 120 min intestinal (0.51 vs. 0.57 and 0.42 log reductions, respectively) phases (Figure 5B). For TV in strawberry smoothies, the mean log virus reduction did not exceed ~0.07 log and was not significantly different between the oral, gastric, or intestinal phases (Figure 5C).

Two-way ANOVA analyzing time (2, 10, 15, 60, and 120 min) and virus matrix (water and blueberry or strawberry smoothies) as two factors revealed that both these factors exerted a significant effect on the overall variation in TV log reduction data, explaining 7.1 and 31.5% of the variation, respectively.

## 4. Discussion

Globally, berries have been associated with multiple foodborne virus outbreaks [12]. However, a recent human volunteer study suggested that consumption of berries contaminated with relatively low levels of HuNoV (<120 genomic copies/g) did not result in clinical symptoms among healthy adults [22]. Because human challenge studies are expensive and difficult to conduct, there is a knowledge gap concerning the association of HuNoV on berries, its survival through the gastrointestinal tract, and the resulting host infections leading to foodborne outbreaks. In this study, we applied the standardized INFOGEST simulated in vitro digestion model to understand the level of inactivation of infectious HuNoV surrogate, TV, as it passes through the different stages of digestion while being on berries. The novelty in our study is that it was performed using standardized digestive fluids supplemented with digestive enzymes and bile, with sequential phases of digestion and two types of berry matrices. In contrast, previous studies that investigated the effects of vitro digestion on foodborne viruses such as HuNoV or its culturable viruses such MNV and FCV have used various digestive fluid formulations without the addition of digestive enzymes or subjecting the viruses to sequential phases of digestion [27,28,29,30]. For example, several studies used commercially available SGF containing 0.2% (*w*/*v*) sodium chloride in 0.7% (*v*/*v*) hydrochloric acid (pH 1.5) and SIF (pH 7.5) of unknown components [27,28,30]. Results from these studies showed that MNV was completely inactivated in SGF (pH 1.5) within 1 h, while resisting inactivation in SIF (pH 7.5) for up to 6 h of incubation at 37 °C [27,28]. In a different study, HuNoV genomic materials were found to be stable for up to 6 h of incubation at 37 °C in commercial SGF (pH 1.5) and SIF (pH 7.5) [30]. The standardized INFOGEST components for SGF include, in addition to pepsin, components that mimic the gastric contents such as KCl, KH_2_PO_4_, NaHCO_3_, NaCl, MgCl_2_(H_2_O)_6_, (NH_4_)_2_CO_3_, and CaCl_2_ (H_2_O)_2_ [23], which may have different effects on these viruses. A previous study used the standardized INFOGEST digestive fluid formulations to test the effect of these fluids against MNV [23]; however, the authors have used a non-physiologically relevant temperature of 25 °C and performed the incubation period for 24 h [29]. Nevertheless, the authors reported that MNV remained stable for 24 h at 25 °C in SSF (pH 7), SGF (pH 3), and SIF (pH 7) that were not supplemented with the digestive enzymes or bile [29]. Therefore, it is important to use physiologically relevant temperature, digestive fluids, and enzymes in order to understand the fate of enteric viruses during gastrointestinal passage.

Our results showed that under consecutive digestion in standardized fluids supplemented with digestive enzymes, limited inactivation occurred for TV spiked in water during the oral and gastric phases (mean infectivity reduction < 0.5 log) (Figure 5A). The latter is similar to the result obtained with TV spiked in water alone and incubated at 37 °C, showing ~0.5 log reduction within 60 min (Figure 3A). Therefore, the gastric pH, the presence of gastric fluid, and pepsin did not contribute to further reduction in TV infectivity other than what was shown with the effect of time and temperature. Previous studies utilizing commercial biorelevant digestive fluids showed high stability of enteric viruses, such as rotavirus and enteroviruses, in these fluids that were not supplemented with digestive enzymes or used consecutively [31,32]. Interestingly, when the fluids were used consecutively, we observed by the end of the intestinal phase that there was significantly less cumulative inactivation in TV infectivity as compared to the oral phase alone (Figure 5A). This phenomenon requires further investigation into the effect of the individual components of these fluids on TV infectivity. However, for one component, MgCl_2_, which is 2-fold higher in the simulated intestinal fluids than in the simulated salivary fluids, it has been shown previously to lessen the reduction in viral infectivity for hepatitis A virus [33]. Thus, there may be components in the intestinal fluids that reverse or lessen the inhibitory effects of preceding digestive fluids on TV infectivity.

In blueberry smoothies alone or following simulated digestion from oral to intestinal phases, TV infectivity showed limited reduction (mean infectivity reduction ~0.5 log). The latter was similar to the ~0.5 log reduction result that occurred in TV spiked into blueberry smoothies alone and incubated under 37 °C for 60 min or 120 min (Figure 3B. In contrast to TV in water, the ~0.5 log reduction observed in blueberry smoothies undergoing simulated digestion was stable throughout the three phases of digestion. Taken together, these results suggested that the cumulative action of digestive fluids and enzymes did not contribute to further reduction in TV infectivity in blueberry beyond what we already shown from the temperature effect alone. In strawberry smoothies, the mean infectivity titers of TV were stable following in vitro simulated digestion. Time, temperature, or digestion did not have significant effects on TV infectivity in the strawberry matrix. Therefore, strawberries may have protective components that render TV less susceptible to infectivity damaging factors. For example, strawberries were reported to have 2-fold higher magnesium ions than blueberries [34], which may help in reducing the loss in virus infectivity as mentioned above [33]. Another reason behind the higher inactivation of TV inside the blueberry matrix in comparison to that of the strawberries may be due to the presence of certain antiviral phytochemicals [20]. Blueberries contain polyphenols, specifically proanthocyanidins, which have been shown to cause significant virus (such as FCV and MNV) reduction, even when incubated under intestinal digestion using SIF (pH 7.5) [27]. These compounds were previously reported to be ~2-fold higher in blueberries than in strawberries [35]. A third reason may be the lower pH of blueberry smoothies in comparison to strawberry (pH 2.8 vs. 3.2, respectively). Lower pH may explain the higher reduction that occurred in blueberries, because TV has been reported to be more susceptible to inactivation in solutions of pH < 3 but not between pH 3 and 8 [36,37]. Interestingly, strawberries are more often implicated in foodborne viral outbreaks in comparison to blueberries, which is known to be related to multiple factors, including surface morphology and method of harvesting [12]. Subsequently, our results that showed higher stability of TV in the strawberry matrix and following in vitro digestion might at least in part explain why strawberries are more often implicated in foodborne virus outbreaks than blueberries.

## 5. Conclusions

Taken together, our results showed for the first time that a human norovirus surrogate, TV, when ingested on berries can survive the simulated gastric and intestinal conditions of the gastrointestinal tract. The virus was more stable in strawberry smoothies alone or under in vitro digestion than in blueberry smoothies. Our findings suggested that the high stability of infectious human norovirus surrogate, especially in strawberries upon ingestion, may partially explain the association between norovirus and strawberry outbreaks. This highlighted the need for better preventive control and hygiene measures to prevent initial berry contamination with HuNoV.

## Data Availability

The original contributions presented in the study are included in the article, further inquiries can be directed to the corresponding author.
